# Effects of semantic categorization strategy training on episodic memory in children and adolescents

**DOI:** 10.1371/journal.pone.0228866

**Published:** 2020-02-18

**Authors:** Eliane C. Miotto, Joana B. Balardin, Maria da Graça M. Martin, Guilherme V. Polanczyk, Cary R. Savage, Euripedes C. Miguel, Marcelo C. Batistuzzo

**Affiliations:** 1 Department of Neurology, Hospital das Clinicas da Faculdade de Medicina da Universidade de Sao Paulo (HC-FMUSP), Sao Paulo, SP, Brazil; 2 Brain Institute, Hospital Israelita Albert Einstein, Sao Paulo, SP, Brazil; 3 Department of Radiology, HC-FMUSP, Sao Paulo, SP, Brazil; 4 Department of Psychiatry, HC-FMUSP, Sao Paulo, SP, Brazil; 5 Banner Alzheimer’s Institute, Phoenix, AZ, United States of America; Kochi University of Technology, JAPAN

## Abstract

Episodic memory is the ability to learn, store and recall new information. The prefrontal cortex (PFC) is a crucial area engaged in this ability. Cognitive training has been demonstrated to improve episodic memory in adults and older subjects. However, there are no studies examining the effects of cognitive training on episodic memory encoding in typically developing children and adolescents. This study investigated the behavioral effects and neural correlates of semantic categorization strategy training in children and adolescents during verbal episodic memory encoding using functional magnetic resonance imaging (fMRI). Participants with age range: 7–18 years were scanned before and after semantic categorization training during encoding of word lists. Results showed improved memory performance in adolescents, but not in children. Deactivation of the anterior medial PFC/anterior cingulate and higher activation of the right anterior and lateral orbital gyri, right frontal pole and right middle frontal gyrus activation were found after training in adolescents when compared to children. These findings suggest different maturational paths of brain regions, especially in the PFC, and deactivation of default mode network areas, which are involved in successful memory and executive processes in the developing brain.

## Introduction

The ability to learn and recall new information is essential to human development and highly demanded during school-age years in children and adolescents. Episodic memory is a critical system associated with the capacity to learn, store and recall personally experienced and temporally specific events [1–2]. A distributed brain network including regions of the prefrontal cortex (PFC), hippocampus and surrounding cortices support episodic memory and follow different developmental trajectories [3]. The PFC has been associated with executive processes that support distinctive components of learning and memory demonstrated by different neuroimaging and lesion studies [4–9]. There are a number of strategic processes that can improve encoding and retrieval of information, such as semantic categorization strategy (grouping information with common features), and such strategies have been associated with executive processes related to regions of PFC including the inferior frontal gyrus (IFG) and dorsolateral prefrontal cortex (dlPFC) [4–6]. Also, the orbitofrontal cortex (OFC) has been linked with early mobilization of effective behavioral strategies [4,6,10]. The fact that the PFC matures gradually during development could explain the delay in children’s ability, in comparison to adolescents, to apply organizational strategies during learning, encoding and retrieving of new information [8,11,12,13]. In contrast, the medial temporal regions, particularly the hippocampi, show early development maturation and engagement during memory encoding [14–16] and the continued neurogenesis in the dentate gyrus in adulthood suggests a lengthy development [17].

Successful efforts to improve episodic memory performance are found in the adulthood and aging literature, particularly, employing semantic categorization strategy, visual imagery, and verbal association strategies in healthy or neurological population [6,18,19,20,21]. In children and adolescents, most of the studies are focused on cognitive training to improve working memory [22–25]. Despite growing interest in the benefits of cognitive training, little is known about the neural mechanisms by which training influences cognitive abilities. Semantic categorization of information is an encoding strategy known to improve verbal episodic memory, as previously described [4,6,7]. In healthy adults, improved episodic memory performance was demonstrated after a brief period of training with semantic categorization strategies associated with bilateral PFC activation, including the IFG, dlPFC, and OFC [6]. In children, few studies indicated that as early as age 4, they can employ spontaneous memory strategies to enhance their remembering, such as category conceptual clustering over color organization, particularly, at 6 to 7 years old [26–27].

Although the participation of PFC is crucial to encode and retrieve new information, there are no studies investigating the role of this region in episodic memory encoding after cognitive strategy training in typically developing brain. In particular, the effects of semantic categorization strategy on episodic memory in children or adolescents have yet not been explored. This is crucial to understand the effects of such strategy on memory performance in the developing brain and to identify possible differences in brain and behavior mechanisms between children and adolescents. Therefore, the aims of the current study were to investigate: (1) the behavioral effects of semantic categorization strategy training in typically developing children and adolescents, (2) the neural correlates associated with the application of this strategy during episodic memory encoding using functional magnetic resonance imaging (fMRI), and (3) differences in behavioral performance and brain correlates between children and adolescents.

## Materials and methods

### Participants

Twenty-five typically developing children and adolescents recruited from the community, age range: 7–18 years, were included in the study. Initially, 71 telephone calls resulted in 35 possible subjects that were interviewed and evaluated. Thirty-six potential participants were excluded due to inclusion and exclusion criteria, both mentioned below. Finally, of the 35 participants who performed the fMRI procedure, 2 subjects did not finish the study and we had to exclude 8 cases due to movement artifacts. The sample was organized into two groups: adolescents’ group (age ≥ 12, n = 13) and children’s group (age ≤ 11, n = 12). These age groups were selected based on previously identified developmental changes corresponding with transitions in childhood (7–12) and adolescence (12–19) [28–30].

The inclusion criteria were: 1) typically developing children and adolescents aged between 7 and below 18 years old; 2) satisfactory reading fluency. Subjects were excluded if they had: 1) previous head injury, cysts or any brain malformation; 2) history of substance abuse; 3) presence of psychiatric disorder, intellectual dysfunction, learning disability, epilepsy or any other neurological condition; 4) pregnancy or lactation; 5) history of previous or current use of continuous medication; 6) any contraindication to MRI; and 7) excessive movement during the scans (higher than 1.5mm across the run). This study was approved by the local Medical Ethics Committee of the University of São Paulo Medical School and all participants and parents/legal guardians gave their written informed consent after being informed about the details of the procedure.

All participants and their parents were interviewed by the Kiddie Schedule for Affective Disorders and Schizophrenia (KSADS-PL) [31] administered by a trained and experienced psychologist to exclude the presence of mental disorders. The Petersen Puberty Scale was administered to ascertain pubertal status [32]. For cognitive assessment, intellectual functioning was examined by the Wechsler Abbreviated Scale of Intelligence (WASI) [33], and reading status by the National School Achievement Test that evaluates basic school achievement by asking children to read aloud regular and irregular words (TDE) [34]. Their results were evaluated considering time to complete the task and number of errors, including mispronounced words. Finally, the Edinburgh Handedness inventory was administered to assess handedness [35].

### Experimental design

The experiment consisted of two fMRI scanning sessions (pre- and post-training) with a block design paradigm using a previously published verbal episodic memory (VEM) paradigm [4,6,7,10] and adapted for children and adolescents [36]. Subjects were scanned during encoding of visually presented word lists and were instructed to remember as many words as possible. Free recall was carried out inside the scanner, right after the fMRI sequence, and the participants were instructed to recall as many words as they could during one minute.

After the first session, participants were taken to a different room to perform the recognition task and to practice the semantic categorization training for approximately 30 minutes. The training was based on learning and applying the semantic categorization strategy using two different training word lists (see training details below). After training, participants completed the second scanning session, free recall, and recognition on the same day. During this second scanning session, the free recall instruction was the same as the first session: “now, try to remember as many words as possible”, i.e. subjects were not instructed to recall the words into categories.

### Verbal episodic memory fMRI task

**Fig 1:**
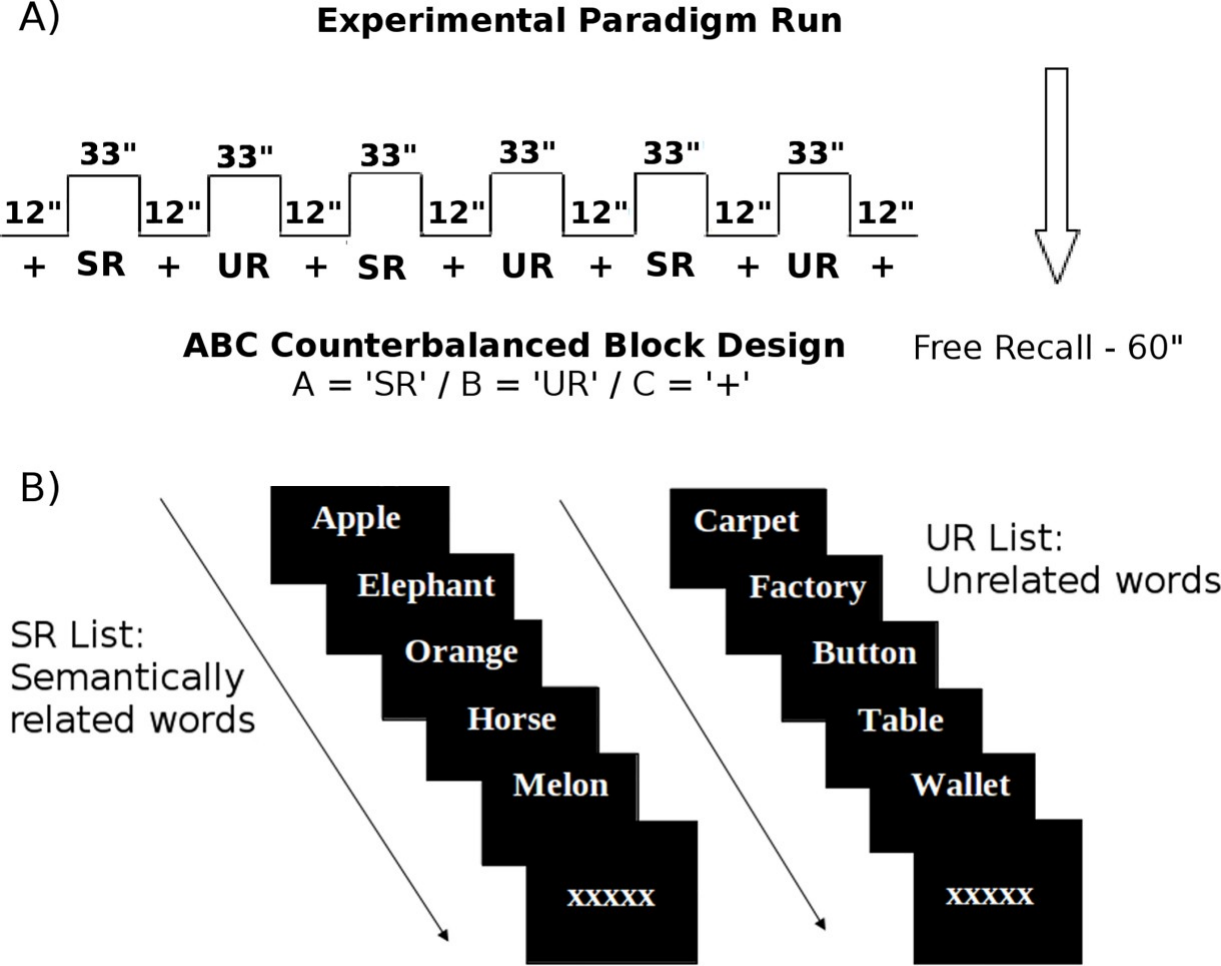
Block design of the verbal episodic memory (VEM) paradigm. A) representation of the ABC experimental paradigm run; B) representation of the encoding lists: semantically related words (SR) and unrelated words (UR).

The fMRI block design VEM paradigm included three conditions: a) a semantically related word list (SR), containing four categories, each one with four words; b) an unrelated word list (UR) and c) a fixation baseline (Fig 1A). Each list included 16 nouns, displayed 1 word at a time, 2 seconds for each word (Fig 1B) and was repeated 3 times in one run, with the word order randomized inside each list–such that two semantically related words could not appear sequentially in the SR list. The fixation baseline between blocks lasted for 12 seconds and included different quantities of “x” and “+” every two seconds. To balance visual stimuli between blocks, the amount of “x” or “+” was defined as the mean number of letters per words in each list.

The order of appearance of the word lists (SR and UR) was counterbalanced across participants to avoid primacy and recency effects. Imaging acquisition was obtained during encoding. The index for measuring the quantity of spontaneous semantic clustering of the SR list was based on the Delis et al. [37] criteria. Briefly, this index was calculated by subtracting an expected score (that depends on the total words of the SR list and the number of words recalled) from the total of semantic clusters (when two words from the same category appear sequentially), ranging from -3 to 9. Outside the scanner, participants performed a yes/no recognition task of 64-items (32 previously seen and 32 new words), where words were balanced for length and concreteness between old and new items. Sixteen of the 32 new words belonged to the same categories of the SR list, in order to increase difficulty. A recognition index was also calculated, ranging from 0 to 1 to summarize overall accuracy performance: index = 1 –[(false positives + false negatives) / (total word lists)].

To avoid ceiling effects, the paradigm had two categories that shared similarities (i.e., fuzzy categories) within the SR list. Categories were: tools, flowers, gardening tools and shoes in the 1^st^ session (pre-training) and fruits, vegetables, clothes and musical instruments in the 2^nd^ session (post-training). During the training session (described below) the first word list included: animals, soft drinks, water animals and sports; and the second-word list, face parts, cheese, cleaning products and body parts.

### Semantic categorization training

In the first scanning session, subjects were not instructed regarding the possibility of semantic organization of the words in the SR list or given any practice with related lists. Therefore, any word grouping by category observed in their subsequent free recall (at the end of the first fMRI acquisition) was presumed to be self-initiated by them. At the end of the first spontaneous scanning session, participants were taken to a different room and given instructions and training on how to apply semantic organizational strategies to new sets of SR word lists. Subjects were instructed to organize the words into categories and to retrieve them accordingly. Children were instructed using an age-appropriate way, for example: “imagine that you are storing this word inside a cabinet drawer; all other words related to this one should go inside the same drawer” and “can you see that ‘lion’ and ‘tiger’ are related? They belong to the same category, ‘animals’ category. Try to organize the next words into groups that belong to the same category”. The training period was carried out within ~30 min or until each participant was effectively able to apply the categorization strategy to the word lists. If a participant failed to apply the strategy to the first training list, a new SR list was presented. All participants were able to learn and apply the semantic strategies within a maximum of two sets of SR lists throughout the training. Immediately after training the application of the semantic categorization strategy, participants were scanned again using the same paradigm as in the first session, except for the use of new word lists, with different categories.

### fMRI data acquisition

Images were collected in a 3 Tesla Achieva Phillips scanner with an eight-channel SENSE head coil. Foam pads were used inside the coil to avoid head movement. E-Prime software 1.0 (Psychology Software Tools Inc., Pittsburgh, PA, USA) was used to present and synchronize the paradigm with the MRI pulse. An LCD visual system: *Eloquence*^*TM*^ *System* for fMRI (Invivo Corp, FL, USA) was used to display the words inside the scanner. Functional images (T2*) were acquired parallel to the anterior-posterior commissural axis, GRE-EPI and compressed sampling was used with sensitivity encoding (SENSE) of 1.2. A whole brain acquisition with 41 axial slices with 3mm thickness and a 0.3mm gap, excitation order: linear down to up, TR 3000ms, TE 30ms, matrix 80x78, FOV 240mm^2^, voxel resolution 3mm^3^ and 90° flip angle. The total time of each run was 4'48'', including 94 volumes–to reach longitudinal magnetization stability of the signal, the first 4 scans were excluded. fMRI sequences were identical for both runs. A T1 high-resolution volumetric sequence (5’58” duration) was also acquired to facilitate further registration of the functional data, with isotropic voxels of 1mm^3^, TR 7.0ms, TE 3.2ms, matrix 240^2^, SENSE 1.5, FOV 240^2^ and a flip angle of 8°.

### Statistical analysis

#### Behavioral data

To test the training effects, within-group comparisons were performed using paired t-test, or Wilcoxon signed rank test, depending on the variables’ distribution, separately in each group. To investigate the influence of semantic clustering on the total recall scores, a linear regression analysis was performed on the number of SR words recalled, also separately for each group. Between-group analyses were performed using independent t-test or Mann-Whitney test to search for performance differences between the groups both, before and after training. Finally, repeated measures ANOVA was conducted to test for the group*training interaction. The adopted alpha level of significance was 0.05, two-tailed, and all behavioral statistical analyses were performed using the JASP, version 0.8.3.1 [38].

#### fMRI data

We used FSL 5.0 (http://fsl.fmrib.ox.ac.uk/fsl, Oxford, UK) [39,40] to perform neuroimaging data processing and statistical analysis. The individual-level analysis included movement correction (MCFLIRT), spatial smoothing (FWHM = 5mm) and spatial normalization to standard space (affine, 12 DoF). Time-series from each voxel were high-pass filtered with a cut-off period of 90 seconds to remove signal drift and low-frequency noise. Subjects were excluded if they presented a relative movement higher than 1.5 mm (one-half of the voxel size), considering translation and rotation. Statistical maps of activity at the individual-level (whole brain analyses) were calculated using the general linear model (GLM), with the FMRIB’s improved linear model (FILM) routines, which is based on semi-parametric estimation of residuals autocorrelation [41]. Each block (SR and UR) was modeled using a boxcar function convolved with a gamma-derived /hemodynamic response function (standard deviation of 3s, mean lag of 6s), and the contrasts 'SR > fixation' and 'UR > fixation' were estimated for each participant. The first contrast was relative to the voxels that were more activated when subjects encoded semantically-related (SR) words in comparison to resting, whereas the second contrast showed the activated voxels when subjects encoded unrelated words (UR) relative to resting.

For higher-level processing, we carried out mean group analyses (average before and after training), within-group comparisons (to test the training effect inside each group), between-group comparisons (to test the age effects–adolescents versus children) and repeated measures analysis of variance (ANOVA), to test the interaction effect between training and age group. All statistical images were conducted using mixed effects variance and both, first-level and higher-level analysis were thresholded by using Gaussian random field-based cluster inference with a threshold of Z > 2.3 at the voxel level and a correction for whole brain multiple comparisons using a cluster significance threshold of P < 0.05.

## Results

### Demographic and behavioral results

Among the 25 participants, 14 were male (56%) and all were right-handed. Puberty developmental stage and years of education are presented in Table 1. Verbal IQ, performance IQ and total estimated IQ from WASI were all within the normal average range. The TDE scores indicated that all participants were literate, and results were within the expected age range (Table 1). When participants were split into two groups, children presented significantly reduced puberty development (t(23) = 3.7, p = 0.001, independent t-test), years of education (t(23) = 5.5, p < 0.001, independent t-test) and TDE-time (t(23) = 2.4, p = 0.026, independent t-test), but no differences were observed on IQ and TDE-errors (Table 1).

**Table 1:** Demographic and clinical characteristics of the sample.

	All Participants (n = 25)	Adolescents (n = 13)	Children (n = 12)	chi-squared / t-test
	n (%) / M (SD)	n (%) / M (SD)	n (%) / M (SD)	p-value
Sex				
Male	14 (56%)	7 (53%)	7 (58%)	1.000ª
PUBERTY DEVELOPMENT					
Age	12.1 (2.3)	13.7 (2.1)	10.5 (1.1)	**<0.001**^**b**^
Petersen's Scale	6.4 (3.9)	8.5 (2.9)	3.8 (3.3)	**0.001**^**b**^
HANDEDNESS				
Right	25 (100%)	13 (100%)	12 (100%)	1.000ª
EDUCATIONAL LEVEL				
Years of Education	6.5 (2.5)	8.2 (1.9)	4.6 (1.3)	**<0.001**^**b**^
Verbal IQ	102.0 (12.9)	101.2 (12.4)	102.9 (14.1)	0.753^**b**^
Performance IQ	96.9 (12.3)	94.9 (10.6)	99.1 (14.1)	0.411^**b**^
Total IQ	99.6 (13.3)	98.0 (11.9)	101.6 (14.9)	0.513^**b**^
TDE time (sec)	105.4 (64.6)	78.9 (37.4)	136.7 (77.1)	**0.026**^**b**^
TDE errors	5.1 (4.8)	3.7 (4.6)	6.7 (4.8)	0.129^**b**^

ª Chi-squared test

^**b**^ Independent t-test

M–mean; SD–standard deviation; IQ–Intelligence quotient; TDE—National School Achievement Test.

Between-group comparisons before training indicated that adolescents were not different from younger children in any free recall memory measure (Table 2). After training, however, adolescents were better than children in almost all free recall variables, particularly for the SR condition, and the SCI: they recalled more words than children and they also made more semantic relations and presented better SCI than children (Table 2). Regarding recognition scores, adolescents presented better performance in almost all indexes, except for the UR index before training (Table 2).

**Table 2:** Between-group comparison of behavioral scores pre and post training.

	Pre						Post					
	Adolescents		Children		t-test / Mann-Whitney		Adolescents		Children		t-test / Mann-Whitney	
	Mean	SD	Mean	SD	*t* / Z	p-value	Mean	SD	Mean	SD	*t* / Z	p-value
FREE RECALL												
Total words recalled	11.2	(5.38)	7.67	(2.93)	1.98	0.059^a^	15.5	(5.01)	7.83	(5.06)	3.78	**0.001**^a^
SR words recalled	6.69	(3.73)	4.50	(2.23)	1.76	0.091^a^	10.7	(2.93)	6.00	(3.88)	3.39	**0.002**^a^
UR words recalled	4.46	(2.60)	3.17	(2.24)	1.32	0.198^a^	4.77	(3.08)	1.83	(1.85)	2.91	**0.009**^a^
Intrusions	0.38	(0.50)	0.08	(0.28)	54.5	0.091^b^	0.31	(0.48)	0.25	(0.45)	73.5	0.810^b^
Perseverations	0.08	(0.33)	0.16	(0.38)	71.0	0.530^b^	0.38	(0.65)	0.17	(0.38)	66.0	0.538^b^
Categories	3.23	(0.72)	2.67	(1.07)	54.5	0.186^b^	3.69	(0.48)	2.67	(1.43)	46.5	0.087^b^
Relations	2.23	(2.58)	1.08	(0.90)	1.45	0.159^a^	5.77	(2.91)	2.50	(2.64)	2.94	**0.008**^a^
SCI	0.99	(1.86)	0.20	(0.55)	1.40	0.174^a^	3.85	(2.50)	1.71	(1.97)	2.44	**0.023**^a^
RECOGNITION												
SR Index	0.89	(0.07)	0.72	(0.09)	5.27	**<0.001**^a^	0.89	(0.06)	0.73	(0.15)	3.53	**0.003**^a^
UR Index	0.87	(0.11)	0.80	(0.07)	1.72	0.099^a^	0.88	(0.09)	0.74	(0.13)	3.24	**0.004**^a^
Total Index	0.88	(0.08)	0.76	(0.06)	4.24	**<0.001**^a^	0.89	(0.07)	0.73	(0.12)	3.97	**0.001**^a^

^a^ Independent t-test

^b^ Mann-Whitney test

SD–standard deviation; SR–semantically related; UR–unrelated; SCI Semantic Clustering index. Statistically significant p-values are highlighted in bold.

Within-group analysis comparing pre-and post-training behavioral performance for each group showed significant improvements after training for adolescents in almost all variables of free-recall and the semantic clustering index (SCI), with the exception of number of intrusions, perseverations and number of UR words (Table 3). On the other hand, within-group analysis for children did not show differences in any free recall measure after training, except for the semantic clustering index (SCI) (Table 3). The recognition scores did not change pre-or post-training in either group (Table 3). Repeated measures ANOVA revealed a significantly group*training interaction for total of words recalled (F(1,23) = 8.25, p = 0.009) and SCI (F(1,23) = 14.0, p = 0.001) (Table 3).

**Table 3:** Within-group comparisons of behavioral scores for adolescents and children separately.

	Adolescents						Children								
	Pre		Post		Paired t-test / Wilcoxon		Pre		Post		Paired t-test / Wilcoxon		ANOVA		
	Mean	SD	Mean	SD	*t* / Z	p-value	Mean	SD	Mean	SD	*t* / Z	p-value	F	p-value	
FREE RECALL															
Total words recalled	11.2	(5.38)	15.5	(5.01)	4.14	**0.001**ª	7.67	(2.93)	7.83	(5.06)	0.17	0.870ª	8.25	**0.009**	
SR words recalled	6.69	(3.73)	10.7	(2.93)	4.95	**<0.001**ª	4.50	(2.23)	6.00	(3.88)	1.33	0.212ª	3.31	0.082	
UR words recalled	4.46	(2.60)	4.77	(3.08)	0.41	0.691ª	3.17	(2.24)	1.83	(1.85)	-1.97	0.075ª	2.59	0.121	
Intrusions	0.38	(0.50)	0.31	(0.48)	-0.47	0.655^b^	0.08	(0.28)	0.25	(0.45)	1.41	0.157^b^	1.29	0.267	
Perseverations	0.08	(0.33)	0.38	(0.65)	1.41	0.157^b^	0.16	(0.38)	0.17	(0.38)	0.00	1.000^b^	1.55	0.226	
Categories	3.23	(0.72)	3.69	(0.48)	2.12	**0.034**^b^	2.67	(1.07)	2.67	(1.43)	0.00	1.000^b^	0.68	0.419	
Relations	2.23	(2.58)	5.77	(2.91)	4.97	**<0.001**ª	1.08	(0.90)	2.50	(2.64)	1.91	0.083ª	4.28	0.051	
SCI	0.99	(1.86)	3.85	(2.50)	4.06	**0.002**ª	0.20	(0.55)	1.71	(1.97)	2.84	**0.016**ª	14.0	**0.001**	
RECOGNITION															
SR Index	0.89	(0.07)	0.89	(0.06)	0.16	0.874ª	0.72	(0.09)	0.73	(0.15)	0.22	0.829ª	0.91	0.350	
UR Index	0.87	(0.11)	0.88	(0.09)	0.50	0.624ª	0.80	(0.07)	0.74	(0.13)	-1.64	0.129ª	0.03	0.868	
Total Index	0.88	(0.08)	0.89	(0.07)	0.45	0.659ª	0.76	(0.06)	0.73	(0.12)	-0.81	0.431ª	2.70	0.114	

ª Paired t-test \

^b^ Wilcoxon test

ANOVA–Analysis of Variance; SD–standard deviation; SR–semantically related; UR–unrelated; SCI—Semantic Clustering index. Statistically significant p-values are highlighted in bold.

Linear regression analysis indicated that the SCI did not predict the number of SR recalled words for both groups before training. However, after training it predicted the number of SR words recalled for children (F(1,11) = 11.9, p = 0.006, β = 1.49, R^2^ = 0.54) and for adolescents (F(1,12) = 18.8, p < 0.001, β = 0.93, R^2^ = 0.60).

Finally, we also conducted another experiment with an independent control group outside the scanner (N = 21, mean age 13.1) to investigate a potential practice effect and the hypothesis that the simple repetition of the experiment would improve the participants’ results. Although we used different word lists in pre and post sections, we cannot rule out eventual benefits that the simple task repetition would cause. This group of children and adolescents was submitted to the same memory task protocol (encoding, free recall and recognition) and the other cognitive tasks (WASI and TDE) but was not instructed to categorize the words or apply any other strategy. Detailed results from this experiment can be seen in the supplementary material (S1 and S2 Tables). Briefly, we found that the practice effect cannot be the explanation for the improvement seen in the total words recalled (ANOVA’s p-value 0.009), SR words (ANOVA’s p-value 0.058) and SCI (ANOVA’s p-value 0.021) in the group that received the training. In other words, the simple repetition of the task does not produce the same effect as the direct and explicit instruction (strategy training) to categorize words.

### Imaging results

Mean activations for the SR and UR conditions before and after training for each group can be found in the (S1 and S2 Figs; S3 and S4 Tables). Briefly, in all adolescent averages we found increased bilateral occipital activation, extending to posterior temporal regions in the left hemisphere, before training. They also showed left inferior frontal and precentral gyri activation for SR condition. After training, a more extensive inferior frontal and pre-central cluster was detected, as well as areas in the medial portion of the superior frontal gyrus (pre-supplementary motor area), bilaterally, and a smaller cluster including the posterior orbital gyrus, the anterior insula and the borders of the parietal sulcus in the left hemisphere (S1A and S1B Fig). For the UR condition, apart from the occipital-temporal clusters in post-training, they also presented activations at the left inferior frontal and precentral gyri (S1C and S1D Fig). Children’s group did not present any activation other than bilateral occipital clusters (S2 Fig) for the SR contrast before training.

**Fig 2:**
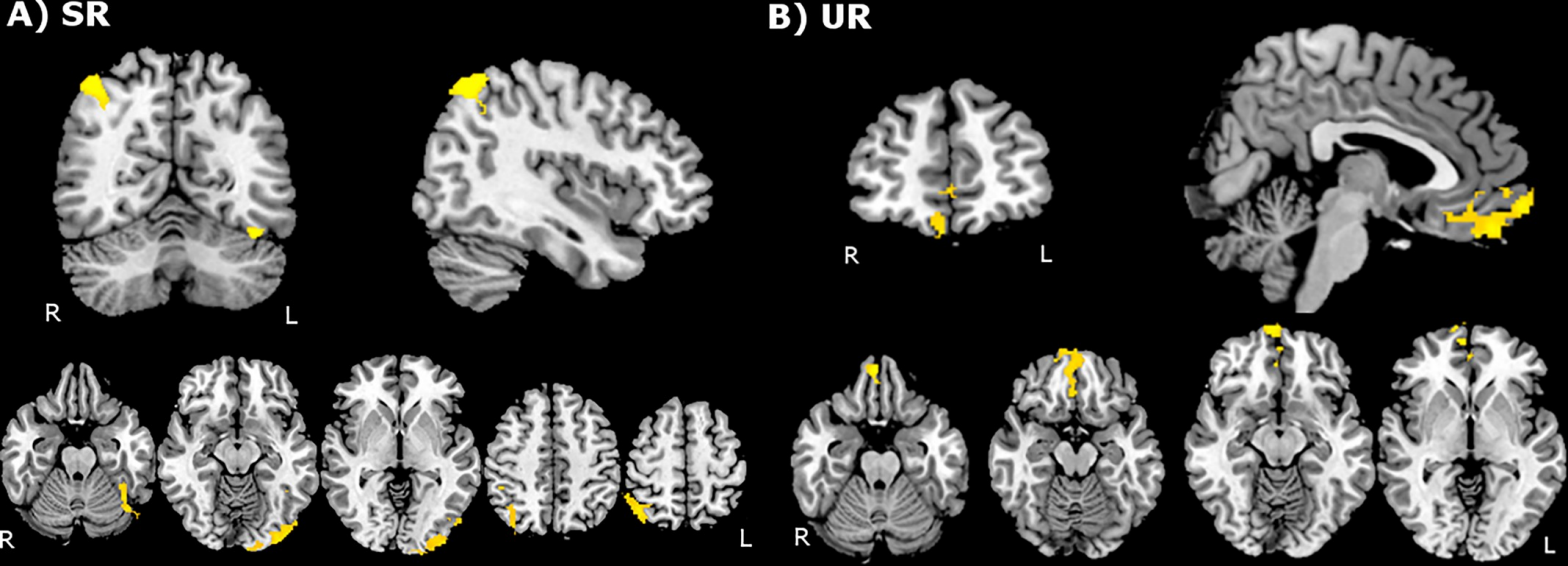
Between-group comparison showing probabilistic activation map before training. A) 'SR' condition. B) ‘UR' condition. Clusters in orange-yellow threshold indicate higher activations in adolescents. Images are shown in radiological orientation. SR–semantically related; UR–unrelated.

**Fig 3:**
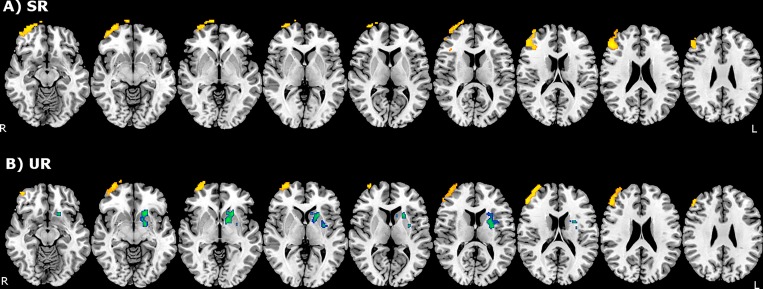
Between-group comparison showing probabilistic activation map after training. A) 'SR' condition. B) ‘UR' condition. Clusters in orange-yellow threshold indicate higher activations in adolescents. Clusters in blue-green threshold indicate higher activations in children. Images are shown in radiological orientation. SR–semantically related; UR–unrelated.

Between-group analysis before training indicated that adolescents presented higher activations in comparison to children for the SR (left occipital lobe, left fusiform gyrus and right inferior parietal lobule) and UR conditions (frontal pole and rectus gyrus) (Fig 2A and 2B, Table 4). After training, between-group analysis revealed that adolescents presented significantly higher activation in the right anterior orbital gyrus, right medial frontal pole and right middle and inferior frontal gyri when compared to children, very similar for the SR and UR contrasts (Fig 3A and 3B, Table 4). Before training, children did not present higher activations when compared to adolescents, and after training a single cluster located in the left basal ganglia was observed for the UR condition.

**Table 4:** Cluster coordinates for activation maps in Figs 2 and 3 –Between-group comparison showing higher activation in adolescents subjects before and after training for SR and UR conditions.

						Coordinates (mm)		
Cluster areas	Hemisphere		Voxels	p-value	Z-MAX	X	Y	Z
**Before training (Adolescents > Children)**								
*SR activation map (Fig 2A)*								
1) Occipital lobe and fusiform gyrus	L	1097		< 0.001	4.52	-42	-64	-20
2) Inferior parietal lobule near the intraparietal sulcus	R	528		0.028	4.29	40	-64	54
*UR activation map (Fig 2B)*								
3) Frontal pole and rectus gyrus	R		633	< 0.001	3.93	4	52	-22
**After training (Adolescents > Children)**								
*SR activation map (Fig 3A)*								
1) Middle and inferior frontal gyri, frontal pole and anterior orbital gyrus	R		805	0.005	3.81	48	36	22
*UR activation map (Fig 3B)*								
2) Middle and inferior frontal gyri, frontal pole and anterior orbital gyrus	R		597	0.021	3.78	52	40	18
**After training (Adolescents < Children)**								
*UR activation map (Fig 3B)*								
1) Caudate nucleus, putamen and adjacent white matter (internal capsule)	L		601	0.020	3.61	-18	18	-8

**Fig 4:**
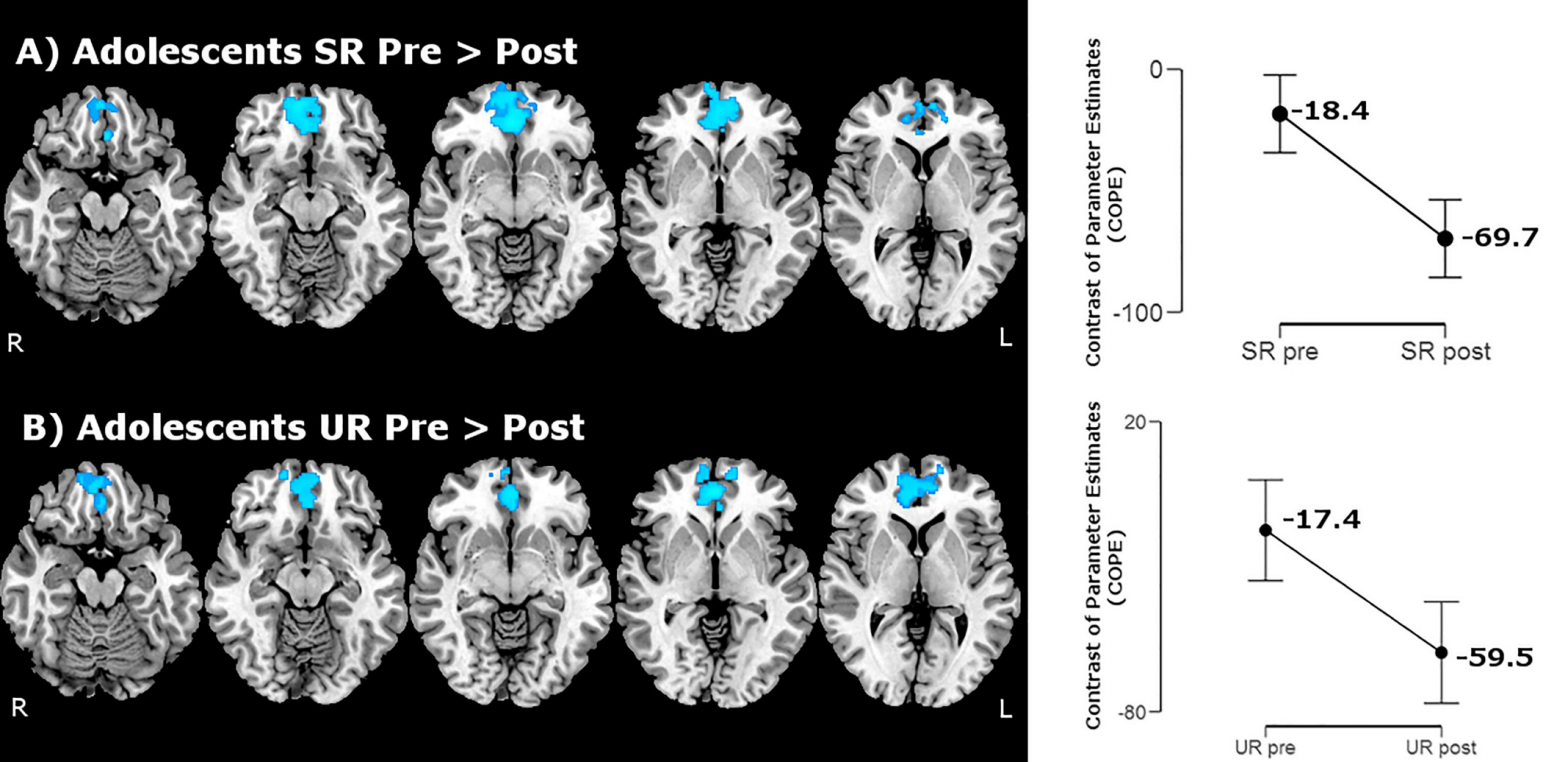
Within-group activation maps for adolescents showing changing in the activation patterns after the training. A) SR contrast and B) UR' contrast. Clusters in light blue indicate decreased activation after the training in the adolescents’ group. Axial images are in radiological orientation.

Within-group comparisons testing training effects in each group indicated that adolescents presented deactivation of medial orbital and rectus gyri, medial frontal pole and anterior cingulate gyrus bilaterally (aMPFC/ACC) for both conditions SR and UR after training (Fig 4A and 4B, Table 5). Children, on the other hand, did not present any difference in the activation pattern (increases or decreases) after training.

**Table 5:** Cluster coordinates for activation maps in Figs 4 and 5: Adolescents within-group analysis and repeated measures ANOVA (group*training interaction).

						Coordinates (mm)		
Cluster	Hemisphere		Voxels	p-value	Z-MAX	X	Y	Z
**Within-group analysis**								
*SR: Pre > Post (Fig 4A)*								
1. Rectus, medial orbital, frontal pole and anterior cingulate gyri	L/R	1087		<0.001	3.66	2	42	-8
*UR: Pre > Post (Fig 4B)*								
2. Rectus, medial orbital, frontal pole and anterior cingulate gyri	L/R	1187		<0.001	3.80	2	46	-2
**Repeated measures ANOVA**								
Group*training interaction–‘SR post and UR pre’ (Fig 5)								
1. Rectus, frontal pole and anterior cingulate gyri	L/R	802		0.003	3.61	12	66	-6

**Fig 5:**
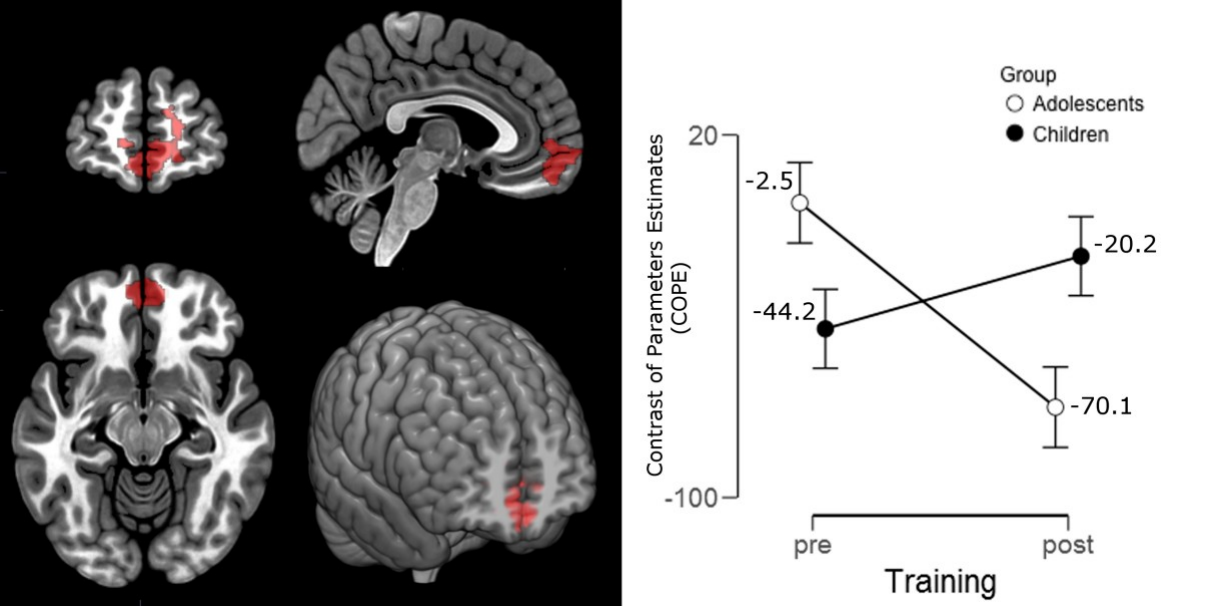
Repeated measures ANOVA group*training interaction effect showing the activation maps for the 'SR post > UR pre' contrast. The cluster is represented graphically in the line chart: adolescents in white and children in black dots. Images are shown in radiological orientation, with exception of the 3D image.

Finally, the ANOVA (group*training interaction) did not reveal any positive cluster for the SR. Then, one final step was conducted: we used the contrast ‘UR pre’ instead of ‘SR pre’ to analyze the group*training interaction. Since both conditions (SR and UR) only differ in terms of the possibility to apply the semantic strategy and it is possible that subjects already clustered words in the SR list before training, we used the contrasts ‘SR post’ and ‘UR pre’ to perform the analysis. In this contrast, a single cluster located in the aMPFC appeared bilaterally, encompassing the frontal pole, the rectus and anterior cingulate gyri (Fig 5 and Table 5). The main effect of time (training outcomes), can be seen in the electronic supplementary materials (S3 Fig and S5 Table). In brief, subjects presented higher activation after training in the left dorsolateral PFC (middle frontal and precentral gyri).

## Discussion

This study investigated the behavioral performance and neural correlates of semantic categorization strategy training in typically developing children and adolescents during verbal episodic memory encoding using fMRI. Episodic memory performance (total number of words recalled, especially in the SR list) and SCI results improved after training and this was largely driven by a better performance of adolescents (which cannot be explained only by practice effect). The neural correlates of the training involved PFC areas and differences between adolescents and children will be discussed below. This study provides novel and important understanding for the neural basis of episodic memory training in children and adolescents.

Before training, between-group comparisons for the SR condition indicated that adolescents showed increased activation in the right inferior parietal lobe, left occipital lobe and fusiform gyrus, and for the UR condition, in the medial frontal pole bilaterally when compared to children. The involvement of these regions before training could be related to the visual and parietal networks implicated in working memory and attention control [42–43].

After training, between-group comparisons demonstrated higher activation in the right anterior and lateral orbital gyri, right frontal pole and right middle frontal gyrus in adolescents for the SR and UR contrasts, consistent with this region’s involvement in strategy selection and/or mobilization found in previous studies [4,6,7,10]. The similar activation found in adolescents for the UR contrast, a condition where the application of the semantic categorization strategy is nearly unfeasible, could be related to the attempt of this group to apply the semantic categorization strategy to the UR word-lists since no previous explanation was provided in terms of the type of lists that were presented during scanning.

These findings suggest that, when compared to children, adolescents show differences in the neural correlates of episodic memory encoding after learning efficient strategies to improve episodic memory performance. After training, adolescents presented better behavioral performance on free-recall and semantic strategy application, particularly for the SR condition, when compared to children. At the same time, in the between-group analyses of neural correlates after training, we observed higher activation of the right anterior and lateral orbital frontal gyri, right frontal pole and right middle frontal gyrus in adolescents. Our results are in line with the concept of prolonged functional maturation of PFC for successful episodic memory encoding in the developing brain in which increasing engagement of PFC is present in adolescence during strategy application [44–46]. In terms of recognition memory scores, all participants (children and adolescents) of the study remembered over 80% of encoded words for both lists (before and after training) indicating that they were engaged to the task inside the scanner.

Within-group comparisons showed bilateral medial orbital and rectus gyri, medial frontal pole and anterior cingulate gyrus (aMPFC and ACC) deactivation for both conditions SR and UR in adolescents but no differences in children. The group*training brain interaction revealed a cluster involving particularly the aMPFC and adjacent areas, mainly driven by the deactivation in these areas found in adolescents after training. The aMPFC and ACC regions are known to be involved in the default mode network (DMN) and several studies demonstrated that a reduction in their activity is necessary for efficient task performance [47–50]. These findings are in line with the significant improvement in behavioral performance found in adolescents after training. Importantly, the ANOVA main effect of time, regarding the training effect in all participants, showed increased activation after training in the DLPFC, replicating previous findings from our group [6–7, 10] and others [4–5] in adults.

There is some evidence for different developmental trajectories of the PFC and the medial temporal lobe related to memory systems in children and adolescents [14–15,44–46,51–52]. One study suggested a delayed functional maturation of prefrontal and parietal cortices for successful encoding of vivid scenes, in contrast to the medial temporal lobe which seems to mature early in childhood for memory encoding of less vivid scenes [15]. Although no previous studies investigated the neural effects of cognitive training using semantic categorization strategies to improve verbal episodic memory in typically developing children and adolescents, our findings corroborate the differences in the developmental trajectories of the PFC for successful episodic memory. The group*training interaction finding at the behavioral and brain level suggested that adolescents showed greater potential training benefits and capacity to learn and apply semantic categorization strategy.

The presence of left IFG activity in adolescents for both SR and UR conditions before and after training supports the process associated with successful memory performance and strategy application. This particular area is known to be engaged in semantic processing, phonological retrieval, and inspection of words [53]. It is also considered as a possible hub in the episodic memory brain network in encoding and associative learning [54]. The ability to apply semantic categorization strategies during verbal encoding has been previously demonstrated in adults to engage similar areas of the PFC [4, 6–7,10]. In one such cognitive training study, with healthy young adults (average age of 38.8 years) using a similar paradigm, participants showed increased activity in the bilateral dlPFC (BA 9/46), inferior PFC (BA 45), and right orbitofrontal (BA 11/47) regions after training associated with a significant improvement in word list recall, in addition to increased use of semantic organizational strategies. The fact that healthy adults recruited more bilateral PFC areas is in line with the prolonged functional maturation of PFC in the age spectrum, when compared to children and adolescents [44–46].

This study should be understood in the context of its limitations and strengths. Regarding its limitations, although we did not use movement as a regressor in our analysis, we had a rigorous criterion to exclude participants that presented an absolute movement higher than 1.5mm, which was half of the voxel size (3mm^3^, isotropic). On average, participants have moved 0.69mm before and 0.73mm after training with no statistical differences in all within-group analyses, including subgroups (S6 Table, *S1 File*). Second, the relatively small sample size of this study may be considered a limitation, and future studies with larger samples should evaluate the neural correlates of semantic training in typically developing participants. Also, it would be interesting to include adult and elderly populations in future studies to compare memory performance and strategy application across the lifespan. Third, we did not control for head size, which could impact in our results, and we used an adult template (MNI 152) to register our images. Fourth, we did not have a control group that went through the same protocol (inside the scan), but we performed a second experiment that minimized the practice effect hypothesis and clearly pointed to the direction that the training had a positive effect on the behavioral outcomes (free recall and SCI). Finally, we instructed subjects off scan on how to apply the semantic clustering strategy in the SR list, although they may also have tried to do it in the UR list post-training scanning. This was the main reason that led us to perform the analysis with the contrast ‘SR post > UR pre’. It is very unlikely that participants would try to semantically categorize unrelated words before training. In regard to its strengths, this is the first study that investigated the effects of semantic categorization training on episodic memory in children and adolescents and showed differences in the neural correlates of memory performance related to PFC areas across typically development. These findings are relevant, not only to the understanding of brain differences in normal maturation and development, but also to the possibility to foster this training strategy to clinical population with episodic memory impairment including neurodevelopmental and acquired brain disorders.

## Conclusion

Episodic memory performance and its brain correlates after semantic strategy training showed different trajectories in typically developing children and adolescents. After training, memory performance significantly improved in adolescents, but not in children, and this improvement was related to deactivation of the anterior medial prefrontal cortex/anterior cingulate and activation of the right anterior/lateral orbital gyri, frontal pole and middle frontal gyrus in adolescents when compared to children. These findings are relevant to the understanding of differences in normal brain maturation and development and to the possibility to apply this cognitive training strategy to clinical population.

## Supporting information

S1 Fig Mean activation maps for adolescents (n = 13) before and after training. A) pre-training SR contrast; B) post-training SR contrast; C) pre-training UR contrast and D) post-training UR contrast. Axial images are in radiological orientation.
(DOCX)

S2 Fig Mean activation maps for children (n = 12) before training for the SR contrast. No cluster survived the statistical threshold after the training for the SR contrast or for any condition of the UR contrast. Axial images are in radiological orientation.
(DOCX)

S3 Fig Mean effect of time (ANOVA), reflecting the higher activation in the post training when compared to the pre training section (n = 25). (DOCX)

S1 Table Demographic, educational and behavioral comparison between the active and control group (baseline scores). (DOCX)

S2 Table Within-group comparisons of behavioral scores for Active training and control group. (DOCX)

S3 Table Cluster coordinates for activation maps in S1 Fig. Average for adolescents before and after training.
(DOCX)

S4 Table Cluster coordinates for activation map in S2 Fig: Average for children before training. (DOCX)

S5 Table Cluster coordinates for activation map in S3 Fig: Mean effect of time. (DOCX)

S6 Table Mean values, within and between and group comparison of absolute and relative movement (mm). (DOCX)

S1 File Behavioral experiment (outside the scanner). (DOCX)
