# Supplementary material for: Effects of semantic categorization strategy training on episodic memory in children and adolescents
Source: PLoS One. 2020 Feb 18;15(2):e0228866. doi: 10.1371/journal.pone.0228866 (PMC7028277; doi:10.1371/journal.pone.0228866)
Supplement: S1 Fig — A) pre-training SR contrast; B) post-training SR contrast; C) pre-training UR contrast and D) post-training UR contrast. Axial images are in radiological orientation. (DOCX) [file pone.0228866.s001.docx]

**Figure S1**

**
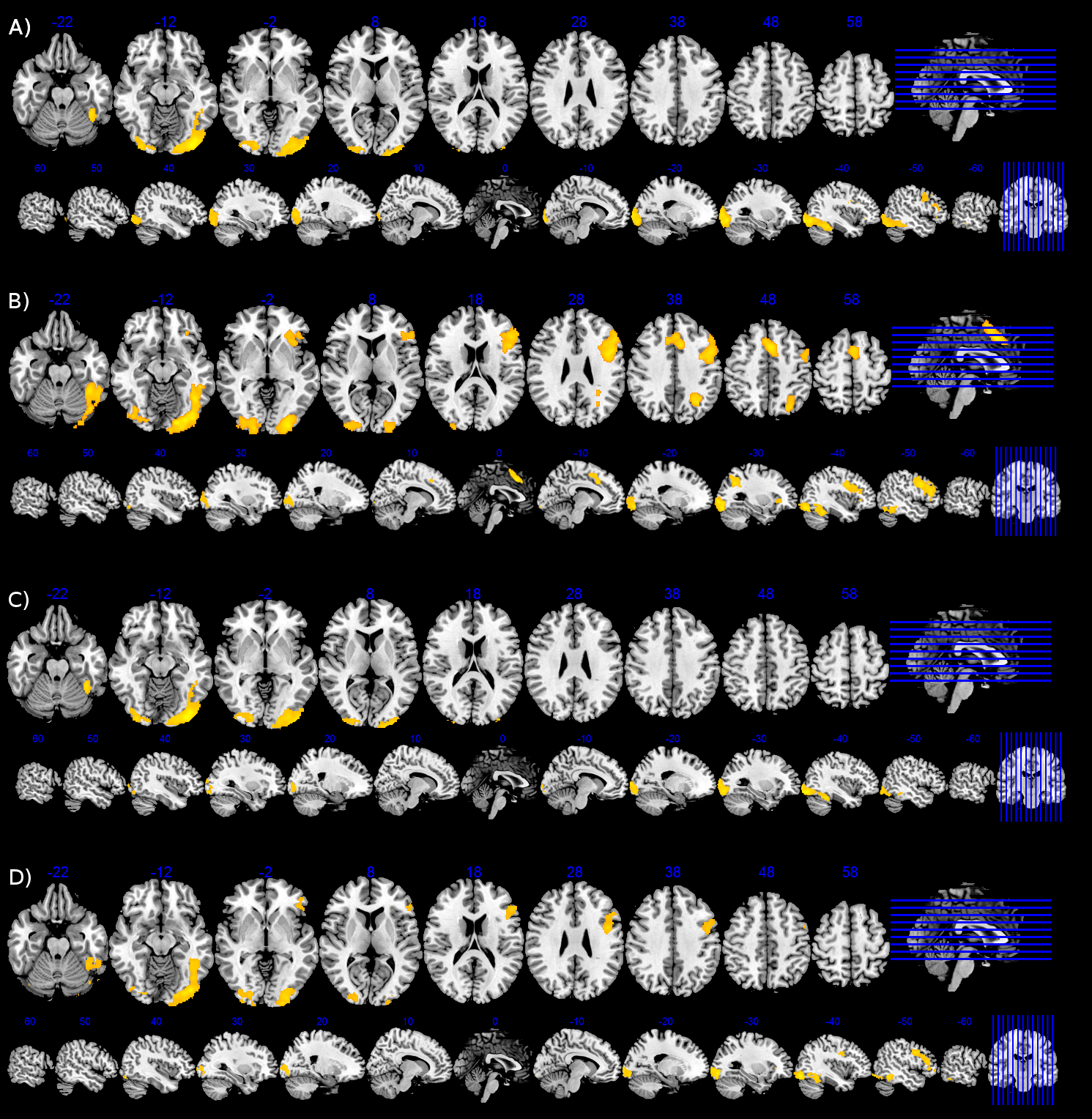
**

Figure S1. Mean activation maps for adolescents (n=13) before and after training: A) pre-training SR contrast; B) post-training SR contrast; C) pre-training UR contrast and D) post-training UR contrast. Axial images are in radiological orientation.
