# Supplementary material for: Effects of semantic categorization strategy training on episodic memory in children and adolescents
Source: PLoS One. 2020 Feb 18;15(2):e0228866. doi: 10.1371/journal.pone.0228866 (PMC7028277; doi:10.1371/journal.pone.0228866)
Supplement: S2 Fig — No cluster survived the statistical threshold after the training for the SR contrast or for any condition of the UR contrast. Axial images are in radiological orientation. (DOCX) [file pone.0228866.s002.docx]

**Figure S2**

**
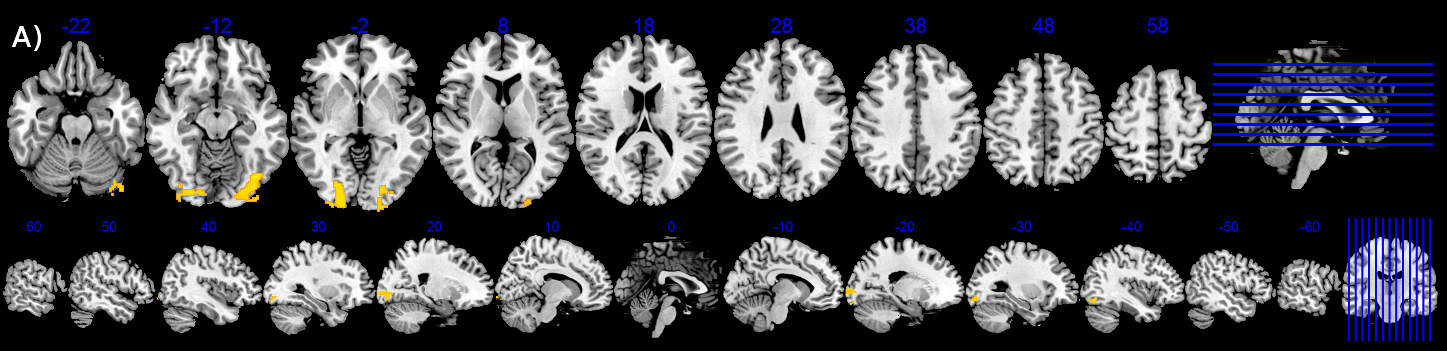
**

Figure S2. Mean activation maps for children (n=12) before training for the SR contrast. No cluster survived the statistical threshold after the training for the SR contrast or for any condition of the UR contrast. Axial images are in radiological orientation.
