# Supplementary material for: Effects of semantic categorization strategy training on episodic memory in children and adolescents
Source: PLoS One. 2020 Feb 18;15(2):e0228866. doi: 10.1371/journal.pone.0228866 (PMC7028277; doi:10.1371/journal.pone.0228866)
Supplement: S3 Fig — (DOCX) [file pone.0228866.s003.docx]

**Figure S3**

**
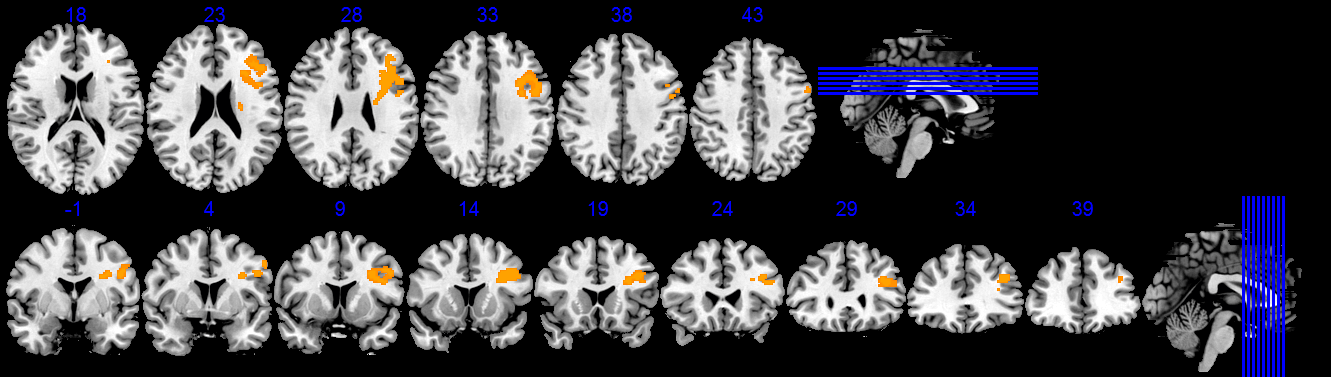
**

Figure S3. Mean effect of time (ANOVA), reflecting the higher activation in the post training when compared to the pre training section (n=25).
