# Supplementary material for: Effects of semantic categorization strategy training on episodic memory in children and adolescents
Source: PLoS One. 2020 Feb 18;15(2):e0228866. doi: 10.1371/journal.pone.0228866 (PMC7028277; doi:10.1371/journal.pone.0228866)
Supplement: S1 Table — (DOCX) [file pone.0228866.s004.docx]

| **Table S1. Demographic, educational and behavioral comparison between the active and control group (baseline scores).** | | | | | |
| --- | --- | --- | --- | --- | --- |
|  | Active training (n=25) | | Control group (n=21) | chi-squared / t-test |  |
|  | n (%) / M (SD) | | n (%) / M (SD) | p-value |  |
| DEMOGRAPHICS |  | |  |  |  |
| Sex (Male) | 14 (56%) | | 12 (%57) | 0.727ª |  |
| Age | | 12.1 (2.3) | 13.1 (2.5) | 0.146^b^ |  |
| Handedness (right) | | 25 (100%) | 21 (100%) | 1.000ª |  |
| EDUCATIONAL LEVEL | | |  |  |  |
| Years of Education | | 6.5 (2.5) | 7.3 (2.4) | 0.268^b^ |  |
| Estimated IQ (WASI) | | 99.6 (13.3) | 94.8 (13.2) | 0.223**^b^** |  |
| TDE time (sec) | | 105.4 (64.6) | 89.2 (27.0) | 0.303^b^ |  |
| TDE errors | | 5.1 (4.8) | 3.0 (2.9) | 0.100**^b^** |  |
| FREE RECALL SCORES | | | |  |  |
| Total words |  | 9.5 (4.8) | 11.1 (3.5) | 0.195**^b^** |  |
| SR words |  | 5.6 (3.2) | 6.1 (2.8) | 0.580**^b^** |  |
| UR words |  | 3.8 (2.5) | 4.9 (2.2) | 0.150**^b^** |  |
| Intrusions |  | 0.2 (0.4) | 0.3 (0.6) | 0.536**^b^** |  |
| Perseverations |  | 0.1 (0.3) | 0.1 (0.3) | 0.794**^b^** |  |
| Categories |  | 3.0 (0.9) | 3.1 (0.8) | 0.495**^b^** |  |
| Relations |  | 1.7 (2.0) | 1.3 (1.5) | 0.459**^b^** |  |
| SCI |  | 0.7 (1.5) | 0.3 (1.0) | 0.193**^b^** |  |
| ª Chi-squared test; **^b^** Independent t-test  Legend: M – mean; SD – standard deviation; IQ – Intelligence quotient; TDE - National School Achievement Test; SR – semantically related; UR – unrelated; SCI Semantic Clustering index. Statistically significant p-values are highlighted in bold. | | | | | |
