# Supplementary material for: Effects of semantic categorization strategy training on episodic memory in children and adolescents
Source: PLoS One. 2020 Feb 18;15(2):e0228866. doi: 10.1371/journal.pone.0228866 (PMC7028277; doi:10.1371/journal.pone.0228866)
Supplement: S2 Table — (DOCX) [file pone.0228866.s005.docx]

| **Table S2. Within-group comparisons of behavioral scores for Active training and control group.** | | | | | | | | |
| --- | --- | --- | --- | --- | --- | --- | --- | --- |
|  | Active training  (n=25) | |  | Control group  (n=21) | |  | Repeated measures ANOVA | |
| FREE | Pre | Post |  | Pre | Post |  |  |  |
| RECALL | M (SD) | M (SD) | p-value | M (SD) | M (SD) | p-value | F | p-value |
| Total words | 9.5 (4.8) | 11.8 (6.3) | **0.009¹** | 11.1 (3.5) | 10.5 (4.2) | 0.364¹ | 7.4 | **0.009** |
| SR words | 5.6 (3.2) | 8.4 (4.1) | **0.001¹** | 6.1 (2.8) | 7.0 (2.7) | 0.168¹ | 3.7 | 0.058 |
| UR words | 3.8 (2.5) | 3.4 (3.0) | 0.371¹ | 4.9 (2.2) | 3.4 (2.7) | **0.020¹** | 1.4 | 0.243 |
| SCI | 0.7 (1.5) | 2.7 (2.5) | **<0.001²** | 0.3 (1.0) | 0.9 (1.7) | 0.064² | 6.7 | **0.021** |

¹ Paired t-test;² Wilcoxon test

Legend: M – mean; SD – standard deviation; SR – semantically related; UR – unrelated; SCI Semantic Clustering index. Statistically significant p-values are highlighted in bold.
