# Supplementary material for: Effects of semantic categorization strategy training on episodic memory in children and adolescents
Source: PLoS One. 2020 Feb 18;15(2):e0228866. doi: 10.1371/journal.pone.0228866 (PMC7028277; doi:10.1371/journal.pone.0228866)
Supplement: S3 Table — Average for adolescents before and after training. (DOCX) [file pone.0228866.s006.docx]

**Table S3. Cluster coordinates for activation maps in Figure S1: average for adolescents before and after training.**

|  |  |  | |  |  |  | Coordinates (mm) | | |
| --- | --- | --- | --- | --- | --- | --- | --- | --- | --- |
|  | Cluster | Hemisphere | | Voxels | p-value | Z-MAX | X | Y | Z |
|  | *SR activation map before training (A)* | | | | | | | | |
|  | 1) occipital lobe and fusiform gyrus | L | 4674 | | < 0.001 | 5.95 | -38 | -94 | -10 |
|  | 2) occipital lobe | R | | 2173 | < 0.001 | 4.62 | 36 | -88 | -10 |
|  | 3) inferior frontal and pre-central gyri | L | | 1018 | < 0.001 | 4.22 | -52 | 8 | 38 |
|  | *SR activation map after training (B)* | | | | | | | | |
|  | 1) occipital lobe and fusiform gyrus | L | 3130 | | < 0.001 | 5.16 | -20 | -90 | -12 |
|  | 2) inferior frontal, posterior orbital and pre-central gyri and anterior insula | L | | 2850 | < 0.001 | 4.96 | -46 | -6 | 34 |
|  | 3) medial superior frontal gyrus (pre-supplementary motor area) | R/L | | 1049 | < 0.001 | 5.27 | 0 | 18 | 44 |
|  | 4) occipital lobe | R | | 1047 | < 0.001 | 4.01 | 34 | -94 | 6 |
|  | 5) borders of intraparietal sulcus | L | | 609 | 0.026 | 4.61 | -30 | -60 | 34 |
|  | *UR activation map before training (C)* | | | | | | | | |
|  | 1) occipital lobe and fusiform gyrus | L | 2806 | | < 0.001 | 5.25 | -34 | -90 | -10 |
|  | 2) occipital lobe | R | | 852 | 0.002 | 2.64 | 32 | -92 | -12 |
|  | *UR activation map after training (D)* | | | | | | | | |
|  | 1) occipital lobe and fusiform gyrus | L | | 2570 | < 0.001 | 7.22 | -40 | -82 | -12 |
|  | 2) inferior frontal and pre-central gyri | L | | 1364 | < 0.001 | 4.28 | -52 | 28 | 16 |
|  | 3) occipital lobe | R | | 734 | 0.006 | 2.2 | 22 | -86 | -10 |
