# Supplementary material for: Effects of semantic categorization strategy training on episodic memory in children and adolescents
Source: PLoS One. 2020 Feb 18;15(2):e0228866. doi: 10.1371/journal.pone.0228866 (PMC7028277; doi:10.1371/journal.pone.0228866)
Supplement: S4 Table — (DOCX) [file pone.0228866.s007.docx]

**Table S4. Cluster coordinates for activation map in Figure S2: average for children before training.**

|  |  | | |  |  |  |  | Coordinates (mm) | | |
| --- | --- | --- | --- | --- | --- | --- | --- | --- | --- | --- |
| Cluster | Hemisphere | | | Voxels | p-value | Z-MAX |  | X | Y | Z |
|  | | *SR activation map before training* | | | | | | | | |
| 1) occipital lobe | L | | 593 | | 0.015 | 3.51 |  | -30 | -86 | -10 |
| 2) occipital lobe | R | | | 477 | 0.047 | 3.55 |  | 18 | -88 | -6 |
