# Supplementary material for: Effects of semantic categorization strategy training on episodic memory in children and adolescents
Source: PLoS One. 2020 Feb 18;15(2):e0228866. doi: 10.1371/journal.pone.0228866 (PMC7028277; doi:10.1371/journal.pone.0228866)
Supplement: S5 Table — (DOCX) [file pone.0228866.s008.docx]

**Table S5. Cluster coordinates for activation map in Figure S3: mean effect of time.**

|  |  |  |  |  | Coordinates (mm) | | |
| --- | --- | --- | --- | --- | --- | --- | --- |
| Cluster | Hemisphere | Voxels | p-value | Z-MAX | X | Y | Z |
| *SR post > UR pre* | | | | | | | |
| 1) middle frontal and precentral gyri | L | 734 | 0.006 | 3.63 | -40 | 18 | 32 |
