# Supplementary material for: Effects of semantic categorization strategy training on episodic memory in children and adolescents
Source: PLoS One. 2020 Feb 18;15(2):e0228866. doi: 10.1371/journal.pone.0228866 (PMC7028277; doi:10.1371/journal.pone.0228866)
Supplement: S6 Table — (DOCX) [file pone.0228866.s009.docx]

|  | | | | | | | | | |  |
| --- | --- | --- | --- | --- | --- | --- | --- | --- | --- | --- |
| **Table S6. Mean values, within and between and group comparison of absolute and relative movement (mm).** | | | | | | | | | |  |
|  |  |  |  |  |  |  |  |  |  |  |
|  | All Participants | |  | Pre | |  | Post | |  |  |
|  | Pre | Post | p-value | Adolescents | Children | p-value | Adolescents | Children | p-value |  |
|  | M (SD) | M (SD) |  | M (SD) | M (SD) |  | M (SD) | M (SD) |  |  |
| Absolute | 0.69 (0.20) | 0.73 (0.31) | 0.267¹ | 0.67 (0.19) | 0.70 (0.21) | 0.717² | 0.74 (0.32) | 0.73 (0.32) | 0.985² |  |
|  |  |  |  |  |  |  |  |  |  |  |
| Relative | 0.14 (0.06) | 0.18 (0.13) | 0.063¹ | 0.13 (0.06) | 0.14 (0.05) | 0.736² | 0.17 (0.16) | 0.18 (0.09) | 0.929² |  |
| ¹ *t* test for paired samples; ² t test for independent samples. | | | | | |  |  |  |  |  |
