# Supplementary material for: Effects of semantic categorization strategy training on episodic memory in children and adolescents
Source: PLoS One. 2020 Feb 18;15(2):e0228866. doi: 10.1371/journal.pone.0228866 (PMC7028277; doi:10.1371/journal.pone.0228866)
Supplement: S1 File — (DOCX) [file pone.0228866.s010.docx]

**Electronic Supplementary Material**

**Behavioral experiment (outside the scanner)**

In this additional behavioral experiment, we collected data from a group of controlled participants (child and adolescents) with a similar study design that was used before, with the only difference that they performed the task outside the scanner. We briefly described here what was conducted: twenty one participants were evaluated in a quiet school room and were assessed with the Wechsler Abbreviated Scale of Intelligence (WASI), the Brazilian National School Achievement Test (TDE), and the memory task, using exactly the same parameters as the scanner section - participants were assessed with E-Prime 2.0 using a HP notebook (ProBook 4430s, i5 intel processor, Windows 7), and saw the same words presented by the same amount of time in the computer screen. Is summary, it was the same task (encoding and recognition), without the training section. Pre and post evaluations had an interval of 30 min between them (the same amount of time as the fMRI study) in which participants performed the cognitive tasks.

Table S1 shows the demographic characteristics of the new sample when compared with the fMRI sample at baseline. As we can see, the two groups were similar in terms of sex, age, handedness and years of education, as well as the cognitive characteristics: IQ and TDE (time and errors). Moreover, before the training these two groups did not differentiate in the memory task performance (immediate recall), as we can see in Table S5. Also, recognition indexes scores were all > 0.78 (total index = 0.84, SR index 0.78, UR index = 0.89), indicating that this new group, as the individuals who were scanned, performed high above the chance level (0.5).

Regarding the training effect (the main reason why we performed this new experiment), Table S2 shows the interaction effect (repeated measures ANOVA) and the within-group comparison (before and after) for both groups: active training and control condition. As we can observe, there was an interaction effect for the total words recalled: while the active group improved the total number of recalled words, the control group did not change significantly their performance. The exact same pattern can be observed in semantic clustering index (SCI) and in the total of semantic related (SR) words recalled, though only a tendency was observed in this last one as the result of the repeated measures ANOVA (p-value = 0.058). No effect was observed in the unrelated words (UR).
